# An Effective Feature Selection Model Using Hybrid Metaheuristic Algorithms for IoT Intrusion Detection

**DOI:** 10.3390/s22041396

**Published:** 2022-02-11

**Authors:** Saif S. Kareem, Reham R. Mostafa, Fatma A. Hashim, Hazem M. El-Bakry

**Affiliations:** 1 Department of Information Systems, Faculty of Computers and Information Sciences, Mansoura University, Mansoura 35516, Egypt; saif.salah.k@gmail.com (S.S.K.); elbakry@mans.edu.eg (H.M.E.-B.); 2 Faculty of Engineering, Helwan University, Cairo 11795, Egypt; fatma_hashim@h-eng.helwan.edu.eg

**Keywords:** Internet of Things (IoT), Gorilla Troops Optimizer, Bird Swarm Algorithm, intrusion detection system, machine learning, feature selection

## Abstract

The increasing use of Internet of Things (IoT) applications in various aspects of our lives has created a huge amount of data. IoT applications often require the presence of many technologies such as cloud computing and fog computing, which have led to serious challenges to security. As a result of the use of these technologies, cyberattacks are also on the rise because current security methods are ineffective. Several artificial intelligence (AI)-based security solutions have been presented in recent years, including intrusion detection systems (IDS). Feature selection (FS) approaches are required for the development of intelligent analytic tools that need data pretreatment and machine-learning algorithm-performance enhancement. By reducing the number of selected features, FS aims to improve classification accuracy. This article presents a new FS method through boosting the performance of Gorilla Troops Optimizer (GTO) based on the algorithm for bird swarms (BSA). This BSA is used to boost performance exploitation of GTO in the newly developed GTO-BSA because it has a strong ability to find feasible regions with optimal solutions. As a result, the quality of the final output will increase, improving convergence. GTO-BSA’s performance was evaluated using a variety of performance measures on four IoT-IDS datasets: NSL-KDD, CICIDS-2017, UNSW-NB15 and BoT-IoT. The results were compared to those of the original GTO, BSA, and several state-of-the-art techniques in the literature. According to the findings of the experiments, GTO-BSA had a better convergence rate and higher-quality solutions.

## 1. Introduction

The Internet of Things (IoT) has emerged in the modern era, pushing the development of new business process technologies through a network of computers and devices capable of communicating and engaging with one another [1]. As the number of cybersecurity attacks on IoT systems rapidly and widely increases, individuals and businesses face a wide range of challenges related to credibility, financing, and business operations [2]. It is possible to characterize cloud computing as a model in which a variety of services and resources are made available to customers on demand, with little involvement from either the service provider or the customer [3]. Most IoT applications in different fields depend on cloud computing to store and process data. Security is a major concern with cloud computing, owing to the massive amounts of data that are stored there. Cyberattacks on cloud computing have increased for several reasons, including the availability and accessibility of hacking tools, which led to the hacker not needing extensive knowledge or exceptional skills to carry out an attack [4].

Consequently, businesses and academia around the world have been paying close attention to the growing need for cybersecurity development. Cyberattacks continue to strike organizations and enterprises despite adopting a variety of security tools such as firewalls, antivirus software, encryption of sensitive data, and biometric verification of end-users [5]. Attackers rely heavily on exploiting system vulnerabilities to gain access to the system and launch various attacks, which may lead to the release of sensitive information. These attacks threaten the confidentiality, integrity, and availability of IoT systems all the time.

Intrusion detection systems (IDS) are among the most effective techniques to protect IoT systems from a wide range of attacks [6,7]. There are several distributed systems that use IDS to detect malicious intrusions and quickly counteract the spread of infection by taking rapid countermeasures [8,9,10]. According to the detection mechanisms, whether anomaly detection or misuse, IDS are classified into two types. Anomaly detection is based on analyzing deviations from usual profile behavior in order to detect harmful actions. A high percentage of false positives (FPs) is the main problem with these IDS, but they are better at detecting innovative sorts of attacks. Misuse detection, on the other hand, can successfully separate genuine from malicious instances based on previously observed patterns [11]. Even though these IDS can reliably detect known attacks, they are unable to detect unknown attacks or variations of known ones.

Machine learning (ML) techniques have been used extensively to improve the performance of IDS [12,13,14], but they have not yet been able to achieve the desired level of accuracy. For IDS to be effective, they must not only be able to distinguish between legitimate and malicious traffic during the process of analyzing network traffic, but they must also be able to determine what type of attack is taking place in the protected system. Another hurdle for ML is the wide range of attack types and network traffic attributes, which widen the problem search area and increase computational and time complexity [15].

Feature selection (FS) is an approach for removing irrelevant and redundant features and picking the best subset of features to improve the definition of patterns belonging to various classes. Based on the use of learning algorithms in the selection process, FS approaches are separated into two categories: wrappers and filters. Filter algorithms evaluate the relationship between a set of features using an independent criterion (such as information measures, distance measures or consistency measures). Wrapper algorithms, on the other hand, use specific learning algorithms to assess the value of a subset of features. In terms of classification accuracy, wrapper techniques outperform filter approaches since the suggested subset of features is directly evaluated using feedback from the learning algorithm [16]. But their performance is highly dependent on the learning process, and therefore they are computationally more expensive than filters.

Another important concern while constructing an FS algorithm is the search for the nearly optimal subset of features. When selecting the best subset of attributes in huge datasets, traditional comprehensive methods such as breadth searches, depth searches, and others are infeasible. Wrapper-based approaches such as neural networks require 2N subsets of a dataset with N features to be produced and assessed [17], which is a computationally intensive operation, especially when evaluating subsets individually. Accordingly, FS is viewed as an NP-hard optimization problem. Its objective function is mainly based on two factors: selecting the minimum number of features while preserving maximum classification accuracy. To deal with this issue, FS is designed either as (1) a multi-objective optimization problem to achieve trade-off solutions between the two contrasting objectives or as (2) a single-objective optimization problem by combining these two objectives, which is often the case in the feature selection literature [18].

Recently, Meta-heuristics (MH) algorithms have shown an excellent performance in a variety of optimization scenarios due to their dynamic search behavior and global search capabilities [19]. It has been widely used in the literature to provide acceptable solutions to FS problems, for example, genetic algorithm [20,21,22], particle swarm optimization (PSO) [23], grey wolf optimizer (GWO) [24,25], harmony search (HS) [26], and the seagull optimization algorithm (SOA) [27]. All MH algorithms, on the other hand, need to balance the exploration and exploitation stages in order to avoid getting stuck in local optima or failing to converge [28]. The solution-seeking process in MH algorithms is plagued with randomness, which is to blame for these issues. This situation necessitates the hybridization of notions from several scientific disciplines. An algorithm with improved performance and accuracy can be created through hybridization, which combines the best characteristics of different algorithms into a single improved method.

Through the investigation of pieces of literature, hybrid algorithms gave better performance than solo algorithms. Still, according to the No Free Lunch (NFL) theorem [29], no algorithm is better than all others in all feature selection problems. Accordingly, in order to deal with feature selection problems more effectively, new algorithms must be proposed or old algorithms must be improved by making some changes to their operators. Therefore, we proposed a novel FS technique based on enhancing the performance of a new metaheuristic algorithm called Gorilla Troops Optimizer (GTO) [30] by utilizing the Bird Swarm Algorithm (BSA) [31]. This proposed hybridization is called the GTO-BSA method, in which BSA is applied to change the search operators of GTO to enhance its performance.

Abdollahzadeh et al. [30] have proposed the GTO as a new optimization method inspired by the social intelligence of gorilla troops in nature. The original GTO authors indicated that GTO is a very competitive algorithm when compared to the other well-known metaheuristic algorithms. However, like other meta-heuristic algorithms, GTO suffers from local optima problem stagnation and premature convergence. Because of this, numerous authors have combined GTO with other methods to increase its performance. For example, Gehad and AboulElla [32] introduced a new version of GTO based on Chaotic Maps (CGTO). This work made a significant contribution by optimizing the convergence performance of the original GTO method using chaos-theory principles. The proposed strategy was evaluated on two different optimization problems: global optimization and multilayer thresholding optimization. The proposal can detect prominent regions compared to the original GTO. In another related work involving GTO, Ahmet Cevahir in [33] demonstrated the outcomes of solving high-dimensional optimization issues using a novel hybrid method called the artificial differential evolution Gorilla Troops Optimizer (ADEGTO). ADEGTO combines the exploratory capabilities of differential evolution (DE) with the GTO algorithm. The performance of ADEGTO was tested on high-dimensional optimization problems, and results revealed that ADEGTO outperformed all other competitive algorithms in terms of solution quality and robustness.

The BSA is a famous metaheuristic algorithm created by Meng et al. [31], and it is inspired by the social behaviors and interactions of bird swarms. In recent years, it has been widely used in various applications, including time-series forecasting [34], regression and clustering [35,36,37], global optimization [38,39,40,41], image processing [42,43,44], and cloud-computing scheduling [45].

In general, the proposed GTO-BSA’s idea is to employ the BSA’s operators as a local search for the GTO, with the main objective of increasing the regular GTO’s performance. GTO-BSA was tested utilizing four IoT-IDS datasets. More importantly, for this study, we compared the GTO-BSA against numerous current metaheuristic algorithms, including the original GTO and BSA, HGS, MVO, HHO, and PSO

Briefly, the following are the study’s key contributions:

- Propose a novel feature selection strategy based on a modified GTO.
- Improve the GTO’s exploitation phase using the BSA algorithm to enhance its convergence.
- Evaluate and compare the proposed GTO-BSA using four IoT-IDS datasets with other current metaheuristic approaches.

The sections of this article’s structure are as follows: Section 2 focuses on earlier studies and related works. Section 3 covers the background. The proposed model is presented in Section 4. Section 5 contains the experimental results as well as a discussion. Finally, Section 6 has the conclusion.

## 2. Related Work

### 2.1. Hybrid Metaheuristics for Feature Selection

This section summarizes recent studies on the subject of feature selection using hybrid metaheuristic (MH) algorithms.

In 1995, Eberhart and Kennedy introduced particle Swarm Optimization (PSO), a swarm intelligence-based evolutionary algorithm [46]. Since then, the PSO algorithm has been employed in numerous studies to solve the feature selection (FS) problem, which was inspired by bird and fish social behavior. The PSO method has various advantages, including its simplicity and high convergence speed. However, this approach has a few drawbacks, such as local optima and population diversity. As a result, a number of articles have combined PSO with other algorithms to increase its performance and use it for FS issues. For example, Moradi and Gholampour [47] suggested a PSO-based hybrid FS method based on a local search strategy. The suggested method, named HPSO-LS, selects the less correlated and salient feature subset using a local search strategy incorporated in particle swarm optimization. The goal of the local search strategy is to use correlation information to assist the particle swarm optimization search process to choose distinctive features. It was compared to 5 current methods for selecting features, and it was truly tested on 13 different benchmark classification datasets. PSO was also used in another work by Mistry et al. [48]. The authors proposed a micro-GA integrated PSO feature selection approach for intelligent face expression detection challenges. A micro-GA was introduced to prevent premature convergence in the original PSO method by using Gaussian mutation in the equation of updating the particle’s velocity. Furthermore, the mechanism for updating velocity depends on the average user’s experience in order to achieve a successful global and local search. Results show that the proposed method outperforms classic GAs and PSO-based feature selection algorithms in identifying face emotions. Zhou et al. [49] used a PSO and a spiral-shaped mechanism (HPSO-SSM) to develop a wrapper-based technique for finding the most relevant and optimum features. The HPSO-SSM made three improvements. First, a series of logistic maps improved the variety-searching process. Second, the original position update algorithm included two new parameters that effectively improved the position quality of the next generation. Finally, a spiral-shaped mechanism was applied as a local search operator in the recognized optimal solution zone. The proposed HPSO-SSM was tested with a kNN classifier, compared with wrapper and filter-based approaches, and evaluated with 20 well-known benchmark datasets.

The Grasshopper Optimization System (GOA) is a new swarm intelligence algorithm inspired by grasshoppers’ natural foraging and swarming behavior. According to a critical study by Mafarja et al. [50], the GOA algorithm and the mutation operator of GA were used to create a new binary hybrid algorithm. The authors used the transfer functions to convert continuous GOA into its binary form. Furthermore, a mutation operator with a reasonable mutation rate was utilized to provide diversified solutions. For 25 benchmark datasets, k-NN analyzed the chosen subset of the features. The categorization accuracy was around 92%, which was higher than that of previous comparison methodologies. GOA and evolutionary operators were combined in the same way by Mafarja et al. [51] to develop the GOA-EPD, which is an upgraded GOA with new evolutionary-based operators for developing an efficient wrapper FS technique. The proposed methods were tested on 22 datasets from UCI. The results showed that the EPD significantly impacted the GOA’s efficacy. Applying the selection mechanism improved the proposed approach’s ability to outperform other optimizers and find the best solutions with superior convergence trends.

Mirjalili et al. [52] developed the Salp Swarm Algorithm (SSA), a modern metaheuristic algorithm that simulates the behavior of salps in deep waters. SSA has been applied as a search strategy in several FS methods [53,54]. This trend of opportunistic search behavior improvement also was seen by Idris et al. [55], who took care of the SSA algorithm’s problems. Using the local search (LS) technique, the researchers enhanced the SSA’s capacity for exploitation. In addition, the study used a chaotic map in conjunction with a novel equation variable to find the best location update strategy for followers. Regarding the feature selection problem, the recommended technique’s efficacy was evaluated on 20 benchmark categorization datasets and 3 Hadith data. Compared to alternative solutions, a dynamic SSA proved to be effective.

Mirjalili [56] proposed the Sine Cosine Algorithm (SCA) for global optimization problems, which uses the features of sine and cosine functions. Neggaz et al. [57] devised a hybrid of SSA and SCA with an extra population diversification mechanism, called a disruption operator. Additional diversity was included to prevent stagnation in the quality of solutions when SSA and SCA were used in conjunction to develop a combined population of potential solution candidates. The results were promising when applied to feature selection problems for datasets with feature sizes ranging from 13 to over 11000. Bharti and Kumar came up with a new SCA-based hybridization [58]. Kumar and Bharti used large-scale datasets to convert conventional PSO to binary variants, then incorporated SCA to better exploration. The researchers used 10 standard benchmark test functions for preliminary testing, then used the k-means technique to solve the clustering problem for seven high- and low-dimensional datasets with from 9 to more than 11,000 features. The proposed method changed the PSO’s velocity equation and embedded the SCA’s position update equation. The PSO’s weighting factor was also adjusted, which was changed in every iteration based on the iteration count. To boost the ability to search long-distance sites, a subset of iterations was chosen to inject the highest inertia weight. The research claimed a considerable enhancement at clustering accuracy comparison with numerous other natural-inspired optimization methods based on statistical *t*-tests. Hans and Kaur [59] presented a new hybrid composition of SCA and Antlion Optimization (ALO). SCA was used to update half of the population, while ALO was used to update the other half. In addition, many random factors were introduced into the position update equations to increase population variety. The authors turned the proposed technique into a binary variation using V- and S-shaped transport functions in feature selection. Experiments were carried out on a total of 18 classification data with a lowest of 9 and a highest of 60 features.

The Mayfly Algorithm (MA) is a newly developed algorithm inspired by mayflies’ flight and mating behavior [60]. For the feature selection challenge, Bhattacharyya et al. [61] proposed combining the Mayfly Algorithm (MA) with a harmony search (HS). The MA acquired many solutions from various search regions and passed them to the HS for further development in this method. As a result, the only purpose of combining HS and MA was to improve the search intensification method. The suggested MA-HS algorithm was tested on 18 classification datasets, proving that the solution quality improved.

### 2.2. Metaheuristic Algorithms for Intrusion Detection

In this section, we highlight several studies that applied metaheuristic algorithms for intrusion detection. Kannan et al. [62] introduced a new intrusion detection model that used a GA-based feature selection approach with an existing fuzzy SVM for effective classification. The method was evaluated on the KDD-Cup 99 database, and it increased detection accuracy while reducing false alarms. The detection accuracy rate for this technique was 98.51%. Murugan et al. [26] suggested a system for enhanced intrusion detection that obtained the best features by using the random harmony search (RHS) optimization method. Restricted Boltzmann Machines were used as classifiers to detect distributed denial of service (DDoS) attacks. Using the 23 best features from the KDD-Cup 99 dataset, the system had an accuracy of 99.92% and a false negative rate (FNR) of 0.11.

Priya et al. [25] suggested the DNN (Deep Neural Network) for IoMT (Internet of Medical Things) networks to recognize and forecast unforeseen cyberattacks to construct a dependable and productive IDS. The suggested DNN framework improved accuracy while reducing computation time by 32%, enabling faster identification of post-intrusion impacts in essential cloud computing. The advancement of networks has always been linked to advances in information technology, but the internet economy is growing due to the Internet of Things. SaiSindhuTheja et al. [27] proposed a denial-of-service detection system that combined the Crow Search Algorithm and opposition-based learning methods into the Oppositional Crow Search Algorithm. The suggested algorithm is used in the feature selection process to detect cyberattacks in the cloud environment. The Recurrent Neural Network (RNN) was used to classify features. The precision, F-measure, accuracy, and recall parameters were used to evaluate the method using the KD-cup 99 dataset. The suggested detection technique exceeded the results of current studies in all four parameters, with a high accuracy of 94.12%, 98.18% precision, 95.13% recall, and an F-measure of 93.56%.

Anjum Nazir et al. [63] introduced Tabu Search–Random Forest (TS–RF), a novel feature selection approach. The TS algorithm executes the attribute search, while the RF approach is used as the learning mechanism in the TS–RF wrapper-based feature extraction technique. The authors used the UNSW-NB15 dataset to test the performance of their model. The accuracy and false positive rate (FPR) were the two most important performance indicators. According to the data, the TS–RF, in conjunction with the RF classifier, achieved an accuracy of 83.12% and an FPR of 3.7%. Although the findings are promising, the authors admitted that they did not consider the UNSW-NB15 dataset’s class imbalance problem.

## 3. Background

### 3.1. FS Problem Formulation

The mathematical modeling of FS is presented in this section. Generally, the classification (i.e., supervised learning) of a dataset has $\;N_{S}\times N_{F}$ dimensions, where $N_{S}$ represents the total number of samples and $N_{F}$ denotes the number of features. The primary goal of the FS algorithm is to select a subset *S* from the total number of features ($N_{F}$), where the dimension of *S* is less than $N_{F}$. The following objective function can be utilized to achieve the subset of features:

(1) $$Fit=\lambda \times \gamma_{S}+\left(1-\lambda \right)\times \left(\frac{\left|S\right|}{N_{F}}\right)$$

where $\gamma_{S}$ denotes the classification error using  S, the selected features are represented by |S|, and λ is applied to maintain the balance between $\left(\frac{\left|S\right|}{N_{F}}\right)$ and $\gamma_{S}$.

### 3.2. Gorilla Troop Optimization

Gorilla Troop Optimization (GTO) is a new metaheuristic technique inspired by gorillas’ group behavior, in which five methods are simulated. Here, a specific mathematical mechanism is presented, with a full description of both the exploration and exploitation phases. In the exploration phase, three techniques are applied. The first is transmigration to an unknown place to increase the exploration of the GTO algorithm, the second technique involves transferring to another gorilla, and the GTO algorithm’s third technique is efficient transmigration across a known location to expand exploration. Two techniques are utilized in the exploitation procedure: observing the silverback and competing for mature females [30].

Phase 1: Exploration

In GTO, all gorillas are considered as possible solutions. During each optimizing operation, the best solution is identified as a silverback gorilla. In the exploration phase, three main strategies are used: initialization at an unknown location to increase GTO exploration, migration towards a well-known position to improve the GTO’s ability to search for a range of optimization spaces, and transmigration towards other gorillas. These three strategies work as follows: when rand is less than parameter (*p*), transmigration to an unknown position is chosen. Moreover, if rand ≥ 0.5, the moving towards other gorillas’ strategy is picked, whereas migration to a known place is activated if rand is less than 0.5. All strategies utilized in the exploration process are mathematically described in Equation (2).

(2) $$XY\left(t+1\right)=\{\begin{array}{ll} \left(UB-LB\right)\times r_{1}+LB, & \;\mathrm{rand}\;<p\\ \left(r_{2}-C\right)\times Y_{r}\left(t\right)+L\times H, & \;\mathrm{rand}\;\geq 0.5\\ Y\left(i\right)-L\times \left(L\times \left(y\left(t\right)-XY_{r}\left(t\right)\right)+r_{3}\times \left(Y\left(t\right)-XY_{r}\left(t\right)\right)\right), & \;\mathrm{rand}\;<0.5 \end{array}$$

where *Y*(*t*) denotes the gorilla’s current position vector and XY(t+1) is the position vector of candidate gorilla for the next *t* iterations. Furthermore, each iteration updates the values of $r_{1}$, $r_{2}$, $r_{3}$, and rand. The random values are selected between 0 and 1. For optimization, the parameter (*p*) must be supplied in the range (0, 1) to show the probability of selecting the migration to an unknown position strategy. The parameter $s\;Y_{r}$ and $XY_{r}$ represent one gorilla from the entire population and the vector of the candidate gorilla’s solution that can be assigned at random, respectively. The upper bound and lower bound of the variables are represented by UB and LB, respectively. One of the candidate gorilla’s position vectors is chosen at random and contains the positions updated in each phase. Finally, Equations (3), (5) and (6) are used to derive *C*, *L*, and *H*, respectively.

(3) $$C=F\times \left(1-\frac{It}{MaxIt}\right)$$

(4) $$F=cos\left(2\times r_{4}\right)+1$$

(5) L=C×l

(6) H=Z×Y(t)

(7) Z=[−C,C]

where MaxIt represents the total number of iterations needed to achieve the optimized solution, and Equation (4) is used to calculate *F*. The symbol cos defines the cosine operation and r4 represents a random number in the range (0, 1). Equation (5) is used to calculate *L*, where the random value *I* selects values between −1 and 1. To simulate silverback leadership, we apply Equation (5). Equation (6) is used to determine *H*, while Equation (7) is used to calculate *Z*, where *Z* denotes a random number in the problem in the range (−*C*, *C*). In the final stage of the exploration phase, the cost of all *XY* solutions is computed, and if the cost of XY(t) is lower than the cost of Y(t), the XY(t) solution will return the solution Y(t)  as the best option (silverback).

Phase 2: Exploitation

In GTO, the exploitation phase utilizes two strategies: observing the silverback and competing for adult females. The silverback gorilla is the troop leader, making all the decisions, directing the troop’s activities, and directing the gorillas to food sources. The *W* parameter is used in the exploitation phase and must be specified before starting the optimization process. If *C* ≥ *W* is selected, the silverback procedure is used in Equation (3); however, if *C* < *W* is selected, competition for mature females is used.

Case 1: If *C* > *W* is chosen, the first strategy is used. Equation (8) can be used to represent this behavior mathematically.

(8) $$XY\left(t+1\right)=L\times M\times \left(Y\left(t\right)-Y_{\mathrm{silverback}\;}\right)+Y\left(t\right)$$

(9) $$M={\left({\left|\frac{1}{N}\sum_{i=1}^{N}XY_{i}\left(t\right)\right|}^{g}\right)}^{\frac{1}{8}}$$

(10) $$g=2^{L}$$

In Equation (8), Y(t)  represents the position vector of a gorilla, while the silverback gorilla’s position vector is represented by $Y_{\mathrm{silverback}\;}$. *L* is also computed using Equation (5). In Equation (9), the position vector of each candidate gorilla in *t* iterations is represented by $XY_{i}\left(t\right)$. The total number of gorillas is denoted by *N*. Additionally, *g* and *L* are calculated by using Equation (10).

Case 2: If *C* < *W*, the phase will be reversed. When baby gorillas reach adulthood, they compete ferociously with other males for mature females. Equation (11) can be used to represent this behavior mathematically.

(11) $$XY\left(i\right)=Y_{\mathrm{silverback}\;}-\left(Y_{\mathrm{silverback}\;}\times Q-Y\left(t\right)\times Q\right)\times A$$

(12) $$Q=2\times r_{5}-1$$

(13) A=β×E

(14) $$E=\{\begin{array}{ll} N_{1}, & \;\mathrm{rand}\;\geq 0.5\\ N_{2}, & \;\mathrm{rand}\;<0.5 \end{array}$$

In Equation (11), the best solution is $\;Y_{\mathrm{silverback}}$, while the gorilla’s present position vector is Y(t). *Q* denotes the force of impact and is computed using Equation (12). In Equation (12), $r_{5}$ represents a range of random numbers between 0 and 1. In Equation (13), the level of violence is determined in a conflict by calculating a coefficient vector. *β* represents a parameter in Equation (13) that must be set before starting the optimization process. E is calculated by Equation (14) and is utilized to mimic the consequence of violence on the dimensions of the solutions. The random value between the range 0 and 1 is represented by rand. In the final stage of the exploitation phase, the complexity of all XY solutions is evaluated, and if the cost of XY(t) is less than the cost of Y(t), the XY(t) solution will update the solution Y(t) and give the best option (silverback). The pseudocode for the GTO algorithm is presented in Algorithm 1, which shows how it works.
**Algorithm 1:** GTO Pseudocode.Inputs: Maximum number of iterations *T*, population size *N*, and parameters p and *β*
Outputs: Gorilla’s location and the objective value
Initializing the population of random size $\;Y_{i}$ (*i* = 1, 2, …, *N*)
Determine the suitable parameters of the gorilla
while (terminating condition is not achieved) do
 Use Equation (3) for updating the value of *C*
 Use Equation (5) for updating the value of *L*
 for (all gorillas ($Y_{i}$)) do
  By using Equation (1) update the location of the gorilla
 end for
 The objective values of gorillas are determined
 if *XY* produces better results than *Y*, update them
 Set $\;Y_{\mathrm{silverback}}$ as the best position of the silverback
 for (each gorilla (*Y_i_*)) do
  if (*C* ≥ *W*) then
   Update the gorilla’s location using Equation (8)
  Else
   Update the gorilla’s location using Equation (11)
  End if
 end for
 The objective values of the gorilla are determined
 If New solutions provide better results than previous ones, update those solutions
  Set $\;Y_{\mathrm{silverback}\;}$ as the best location of the gorilla (silverback)
end while
Return YBestGorilla, bestFitness

### 3.3. The Bird Swarm Algorithm

The Bird Swarm Algorithm (BSA) [31], inspired by the social interactions and behavior of swarms of birds, solves optimization problems by simulating feeding, flight, and vigilance compartments. The social behaviors of birds can be boiled down to five simple rules, which are described below:

- Rule 1: There are two possible states for each bird: vigilance and foraging.
- Rule 2: In the foraging mode, each bird keeps track of and remembers its own best experience as well as the best experience within the swarm in terms of food positions. This information will have an impact on its mobility and food search path.
- Rule 3: Each bird competes to move closer to the flock’s center in the vigilance state, assuming that birds with large reserves are closer to the flock’s center. Predators are less likely to attack birds near the center.
- Rule 4: Birds travel from one location to the next, and they alternate between producing and foraging. The algorithm considers that the birds with the most reserves are producers and those with the least are foragers. On the other hand, other birds are randomly labeled as producers or foragers.
- Rule 5: Producers are always on the lookout for new sources of food. The scroungers chase a producer at random in search of food.

The following are the mathematical expressions for the aforementioned rules.

#### 3.3.1. Foraging Behavior

Individuals and groups of birds explore for food according to their own experiences and the collective experience of other birds in the swarm. The following is a mathematical representation of Rule 2:

(15) $$x_{i,j}^{t+1}=x_{i,j}^{t}+\left(p_{i,j}-x_{i,j}^{t}\right)\times C\times \mathrm{rand}\left(0,1\right)+\left(g_{j}-x_{i,j}^{t}\right)\times S\times \mathrm{rand}\left(0,1\right)$$

where j represents a set of uniformly distributed independent numbers in the range (0, 1). The two positive numbers S and C stand for social and cognitive accelerated coefficients, respectively. $P_{i,j}$ is the *i*th bird’s best previous location, and $g_{j}$ is the previous best shared position of the swarm.

#### 3.3.2. Vigilance Behavior

Given Rule 3, each bird will attempt to proceed to the middle of the swarm and engage in surveillance behavior; as a result, each bird will not accelerate to the middle of the swarm immediately. This can be stated as follows:

(16) $$x_{i,j}^{t+1}=x_{i,j}^{t}+A1\left({\mathrm{mean}}_{j}-x_{i,j}^{t}\right)\times \mathrm{rand}\left(0,1\right)+A2\left(p_{k,j}-x_{i,j}^{t}\right)\times \mathrm{rand}\left(-1,1\right)$$

(17) $$A1=a1\times \exp\left(\frac{-pFit_{i}}{\;sumFit+\varepsilon }\times N\right)$$

(18) $$A2=a2\times \exp\left(\left(\frac{pFit_{i}-pFit_{k}}{\left|pFit_{k}-pFit_{i}\right|+\varepsilon }\right)\frac{N\times pFit_{k}}{sumFit+\varepsilon }\right)$$

where *k* denotes a positive number between 1 and *N* that is chosen at random. The best fitness value at the *i*th position is $pFit_{i}$ and sumFit is the total of the swarms’ best objective values. ε is utilized to ignore the zero-division error. The place of the jth average element of the entire swarm is the mean *j* In this expression, a1 and a2 represent the positive constant values (0, 2). Given that all birds desire to be in the middle of the swarm, the outcome of rand (0, 1) and A1 should not exceed 1. When a bird travels towards the swarm’s center, A2 is utilized to imitate the immediate effect caused by a specified interference.

#### 3.3.3. Flight Behavior

Given Rule 4, birds can fly to a different location in reaction to predation risks, for foraging or for any other purpose. The birds will resume their search for food at the new location. The scroungers try to consume food found by the producers, while the producers hunt for food patches. The following are examples of producers’ and scroungers’ behavior:

(19) $$x_{i,j}^{t+1}=x_{i,j}^{t}+\mathrm{rand}n\left(0,1\right)\times x_{i,j}^{t}$$

(20) $$x_{i,j}^{t+1}=x_{i,j}^{t}+\left(x_{k,j}^{t}-x_{i,j}^{t}\right)\times FL\times \mathrm{rand}\left(0,1\right)$$

To simplify, we suppose that each bird moves towards another position in the unit interval *FQ*. Here, *FQ* denotes a positive number. Algorithm 2 presents the pseudocode for the BSA algorithm and demonstrates how it works.
**Algorithm 2:** Bird Swarm Algorithm (BSA) Pseudocode.**Input:** *N*: number of birds (individuals) in the given population *M*: maximum iterations of the algorithm *FQ*: the frequency of flight behavior by individuals *P*: the foraging probability of finding food
Define five constant values: *S*, *C*, *FL*, *a*1, *a*2
*t* = 0 for initializing the population size and determining the relevant variables
Approximate the fitness value of N individuals or birds, and obtain a robust solution
While (*t* < *M*)
 If (*t* % *FQ* ≠ 0)
  For *i* = 1:*N*
   If rand ≤P
    Use Equation (15) for birds foraging for food
   Else
    Use Equation (16) for birds undertaking surveillance
  End if
  End for
 Else
 Producers and scroungers: The swarm is divided into two sections:
 For i=1:N
  If i=producer
   Equation (19) determines “producing”
  Else
   Equation (20) determines “scrounging”
  End if
 End for
End if
 Determine new solutions
 If better results are obtained using new solutions than the previous ones, replace them
 Obtain the ongoing best solution
 t = t + 1
End while
**Output:** A bird or individual from the given population with the best fitness function

## 4. Proposed Hybrid GTO-BSO for Feature Selection

This section explains the structure of the proposed GTO-BSA method, which combines both the GTO and BSA algorithms. Like any metaheuristic, GTO suffers from a balance between exploitation and exploration, which leads to it being trapped in a local optimum. This section describes our proposed hybrid GTO-BSA to tackle this weakness. To enhance the global searching and local searching abilities, we propose a novel improvement of the GTO algorithm using four strategies: (1) the control randomization (CR) parameter and (2) an advanced nonlinear transfer function to balance exploration and exploitation, (3) different settings in the GTO exploration phase, and (4) a novel local updating position strategy based on the BSA algorithm.

- Strategy 1: Control randomization (CR) parameter.

Fine-tuning of randomization parameters ensures the search algorithm’s good behavior and plays a vital role in achieving the balance between the exploration and exploitation phases. The GTO and BSA algorithms are missing this randomization control. In this study, a control randomization (CR) parameter is presented that generates a variable number between positive and negative values. This allows a good scan of the given search space and avoids stagnation in the sub-local optimal solution. CR is given by:

(21) CR=2×rand−1

- Strategy 2: Advanced nonlinear transfer function

The transfer function plays a major role in transferring the search algorithm between the exploration and exploitation phases; the success of balancing between them is affected by the behavior of the transfer function. The linear transfer function fails in achieving this balance in many problems because it controls the behavior of the algorithm during iterations as follows: (a) first half of the iterations for the exploration phase and (b) second half of iterations for the exploitation phase only. Therefore, it is a good idea to switch between both phases during iterations so that the algorithm can get out of suboptimal areas if they get stuck in it during iterations.

In the proposed GTO-BSA method, the linear transfer function was replaced by a new one that prevents it from struggling into a local optima since the old transfer one may be trapped in the local region during the last half of iteration (as it totally used exploitation equations). Therefore, an advanced nonlinear transfer function (*NF*) is proposed that transfers between the exploration and exploitation phases properly. *NF* is given by:

(22) $$NF=sin\left(\varphi -\frac{it}{MaxIt}\right)$$

where φ is a constant (≥1), while it and MaxIt are the current iteration and total number of iterations, respectively.

- Strategy 3: Different settings of the GTO exploration phase.

The exploration phase of the GTO algorithm depends on visiting random locations to explore the given region. As demonstrated in Equation (2), the GTO algorithm has three strategies in the exploration phase. Here, we simplify the exploration phase by using only one strategy of the GTO exploration phase to decrease the time demand and, at the same time, to enhance it to satisfy the requirements of good exploration behavior. The original GTO’s third exploration strategy is calculated by Equation (2).

In the proposed GTO-BSA method, this equation is modified by (1) control randomization using CR in Equation (2), (2) removing the random parameter $r_{3}$, and (3) NF is added in Equation (22) is added to achieve the balance as described above. Accordingly, the proposed exploration phase is as follows:

(23) $$XY\left(t+1\right)=Y\left(t\right)-CR\times NF\times L\times \left[L\times \left(Y\left(t\right)-XY_{r}\left(t\right)\right)+\left(Y\left(t\right)-YX_{r}\left(t\right)\right)\right]$$

- Strategy 4: Propose a new exploitation phase based on the BSA algorithm.
  The GTO exploitation phase consists of two strategies: observing the silverback and competing for mature females.
  ○ The first strategy is demonstrated in Equation (8), in which the silverback guides the agents without any deviation, which is good exploitation behavior. However, this equation does not contain a randomization control parameter, which is important in all phases of the metaheuristic algorithm. Accordingly, this equation is enhanced as follows:
    (24) $$XY\left(t+1\right)=Y\left(t\right)+CR\times NF\times L\times \left|Y\left(t\right)-Y_{\mathrm{silverback}}\left(t\right)\right|$$
    where NF is a nonlinear transfer function that is used to gradually move the agents toward the global optimum location. The parameter (M) is removed from the original equation, as it may deviate the agent from the best location; also in addition, the absolute value is taken so as not to affect the control randomization and NF parameter.
  ○ The second strategy is illustrated in Equation (11). This equation simulates competition between adult gorillas for mature females. It is obvious from this equation that the agents are guided by any unknown location that results from multiplying the best position by the variable Q, which can affect the convergence speed. To avoid this issue, we introduce a new local strategy to the GTO to improve the exploitation performance of the GTO through the BSA algorithm. This improvement combines the updating technique of the BSA (Equation (16)) into the structure of the GTA. The aim of this step is to add more flexibility to the GTA algorithm when exploring the search domain and improving its diversity, which helps in quickly reaching the optimal value.

**Figure 1:**
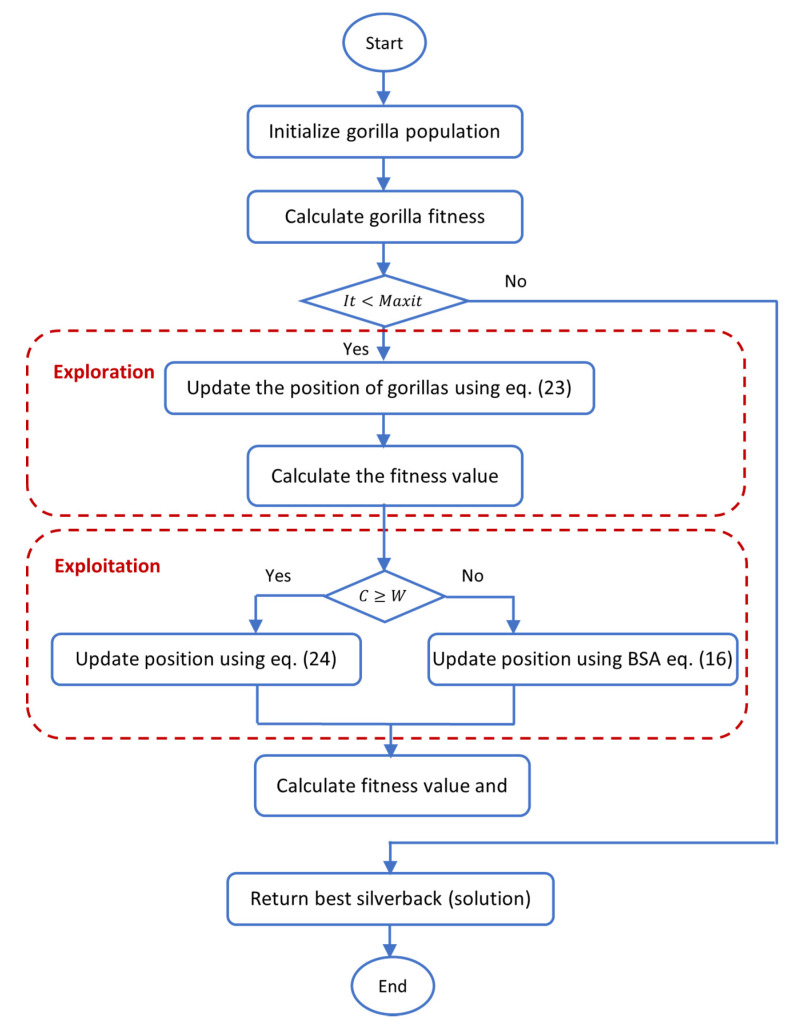
Flow chart of the proposed Gorilla Troops Optimizer (GTO)-Bird Swarm Algorithm (BSA).

The flowchart and pseudocode of the proposed GTO-BSA method were illustrated in Figure 1 and Algorithm 3, respectively.
**Algorithm 3:** Proposed GTO-BSA Pseudocode.**Inputs:** Maximum number of iterations *T*, population size *N*
**Outputs:** Gorilla’s location and the objective value
**% Initialization%**
Initialize the population of random size $\;Y_{i}$ (*i* = 1, 2, …, *N*)
Determine the suitable parameters of the gorilla
**%Main Loop%**
while (terminating condition is not achieved) do
 Use Equation (3) for updating the value of *C*
 Use Equation (5) for updating the value of *L*
  **% Exploration Procedure%**
 for (all gorillas ($\;Y_{i}$)) do
  By using Equation (23), update the location of the gorilla
 end for
 The objective values of gorillas are determined
 if *XY* produces better results than *Y*, update them
 Set $\;Y_{\mathrm{silverback}}$ as the best position of the silverback
  **% Exploitation Procedure%**
 for (each gorilla ($\;Y_{i}$)) do
  if (*C* ≥ *W*) then
   Use Equation (24) to update the gorilla’s location
  Else
   Use Equation (16) to update the gorilla’s location (BSA algorithm)
  End if
 end for
 The objective values of the gorilla are determined
 if new solutions provide better results than previous ones, update those solutions
  Set $\;Y_{\mathrm{silverback}\;}$ as the best location of the gorilla (silverback)
end while
Return YBestGorilla, bestFitness

### Computational Complexity of GTO-BSA Method

Computational complexity is one of the most important aspects to consider when evaluating the performance of an algorithm. The computational cost of the proposed approach can be gleaned from a variety of factors, including the size of the population, the maximum number of iterations, problem dimensions, and the updating mechanism. GTOBSA is a hybrid algorithm that combines both GTO and BSA. Therefore, computational complexity is O(GTOBSA)=O(GTO)+O(BSA).

O(GTOBSA)=O(initialization)+T×(O(evaluation)+O(Updating)

- The computational complexity of initialization: O(N×D)
- The computational complexity of evaluation of an individual: O(N)
- Updating process can be calculation as follows O(T×N×D)

Accordingly, the computational complexity of the proposed GTO-BSA algorithm can be expressed as follow:

O(GTOBSA)=O(ND)+T×(O(N)+O(ND))=O(T×N×D)

where T is the maximum number of iterations, N is the population size, and D is the problem dimension.

## 5. Experimental Results and Discussion

In this section, the quality of the proposed GTO-BSA method to determine the optimal subset of features is assessed by comparing it with other metaheuristic feature selection algorithms, including the original GTO [30], BSA [31], HGS [64], MVO [65], HHO [66], and PSO [23]. All these algorithms are applied to four IoT intrusion datasets. These algorithms have the same parameter setting as defined in Table 1. Moreover, the K-nearest neighborhood (K-NN) is used as a classifier. The experiment was performed on an Intel(R) Core (TM) i7-6700HQ CPU (2.60 GHz; 2.59 GHz with 64 GB). In the Windows 10 operating system, the programming environment is MATLAB R2021b.

### 5.1. Description of the Datasets

The proposed model was tested using four datasets: NSL-KDD, CICIDS-2017, UNSW-NB 15, and Bot-IoT Dataset. Most researchers use these datasets to evaluate the performance of proposed systems.

#### 5.1.1. NSL-KDD Dataset

The NSL-KDD dataset was proposed as a solution to some of the KDDCUP’99 dataset’s fundamental issues. The NSL-KDD has the following advantages over the original KDD dataset: It removes redundant data from the train set, ensuring that classifiers are not skewed toward more common records. The NSL-KDD dataset contains 41 features and 5 classes (normal and 4 forms of attack: Dos, Probe, R2L, and U2R) (Table 2) [67,68].

#### 5.1.2. CICIDS-2017 Dataset

The Canadian Institute for Cybersecurity (CIC) released the CICIDS-2017 dataset, covering common benign and up-to-date attacks. It also includes the results of a network traffic analysis performed by CICFlowMeter, which included flows labeled on the basis of the date, the source and destination IPs, the source and destination ports, protocols, and assaults. It is one of the most recent intrusion-detection datasets, and it includes current attacks, including DDoS, brute force, XSS, and SQL injection attacks (Table 3). This dataset has 2,830,743 records on 8 files, with each record including 78 distinct features with their label [69].

#### 5.1.3. UNSW-NB15 Dataset

The UNSW-NB15 dataset has been created by extracting a combination of everyday normal and contemporary network traffic attack activities using an IXIA PerfectStorm tool. Using the tcpdump utility (pcap files), 100 GB of raw network traffic was captured. Each pcap file is 1000 MB in size to make packet analysis easier. In a parallel implementation, the Argus and Bro-IDS methodologies and 12 procedures were used to create 49 features with the class label. There are 2,540,044 records in this dataset, which are housed in 4 CSV files. A portion of this dataset was also separated into a training and testing set. The training set consisted of 175,341 records, whereas the testing set included 82,332 records from all attack kinds and standard records. The UNSW-NB15 dataset’s associated attacks were divided into nine categories as follows: Fuzzers, Analysis, Backdoor, DOS, Exploit, Generic, Reconnaissance, Shellcode, and Worm [70].

#### 5.1.4. BoT-IoT Dataset

The Bot-IoT [71], dataset was created in the Cyber Range Lab of UNSW Canberra Cyber using Industry IoT (IIoT) smart-home equipment to collect IIoT traffic samples. Thermostats, motion-activated lighting, garage door openers, refrigerators and freezers, and weather-monitoring systems are examples of smart IIoT products. The data are available in two formats: the complete version, which comprises over 72 million entries, and the 10% version, which contains over 3.6 million records (Table 4).

Note: The proposed GTO-BSA method will be tested on 5% of the full dataset in our experiments.

### 5.2. Performance Measures

To test the proposed GTO-BSA approach, we used a variety of evaluation methods. Here, the confusion matrix is explained in detail, where *TP* and *TN* stand for true positive and true negative, respectively; the abbreviations *FP* and *FN* stand for false positive and false negative, respectively. These are metrics used to evaluate a classifier’s accuracy, specificity, and sensitivity [27].

(25) $$\mathrm{Accuracy}=\frac{TP+TN}{TP+FP+FN+TN}$$

(26) $$\mathrm{Sensitivity}\text{\{}\mathrm{recall}\text{\}}=\frac{TP}{TP+FN}$$

(27) $$\mathrm{Specificity}=\frac{TN}{TN+FP}$$

- Average accuracy ($AVG_{Acc}$): This metric is used to calculate the rate at which data are classified correctly. Because each procedure is repeated 30 times (*Nr* = 30), the $AVG_{Acc}$ is calculated as follows:
  (28) $$AVG_{Acc}=\frac{1}{N_{r}}\sum_{k=1}^{N_{r}}Acc_{Best}^{k}$$
- The average fitness value ($AVG_{\mathrm{Fit}\;}$) is used to evaluate the performance of an applied method, and it is calculated using the following equation to calculate the classification error rate and reduce the selection ratio.
  (29) $$AVG_{\mathrm{Fit}}=\frac{1}{N_{r}}\sum_{k=1}^{N_{r}}\;\mathrm{Fit}{\;}_{\mathrm{Best}}^{k}$$
- The average number of features chosen ($AVG_{\left|fs_{\mathrm{Best}\;}\right|}$): This measure is used to determine the ability of a method to minimize the number of features in an overall number of runs, and it is calculated as follows:
  (30) $$AVG_{\left|fs_{\mathrm{Best}\;}\right|}=\frac{1}{N_{r}}\sum_{k=1}^{N_{r}}\left|fs_{\mathrm{Best}\;}^{k}\right|$$
- Average computation time ($AVG_{\mathrm{Time}\;}$): This metric is used to find the average CPU time(s), as shown in the equation below.
  (31) $$AVG_{\mathrm{Time}}=\frac{1}{N_{r}}\sum_{k=1}^{N_{r}}\;\mathrm{Time}{\;}_{\mathrm{Best}}^{k}$$
- Standard deviation (*STD*): *STD* is used to evaluate each method’s quality and analyze the data obtained in multiple runs. It is calculated as follows:
  (32) $$STD_{Y}=\sqrt{\frac{1}{N_{r}}\sum_{k=1}^{N_{r}}{\left(Y_{\mathrm{Best}\;}^{k}-AVG_{Y}\right)}^{2}}$$

Note: $STD_{Y}$ is calculated for each of the following metrics: accuracy, fitness, time, number of selected features, sensitivity, and specificity.

### 5.3. Results and Discussion

The experimental results are presented and discussed in this section. The proposed GTO-BSA was evaluated on four well-known IoT-IDS datasets. The performance of the proposed GTO-BSA was compared with six algorithms: GTO, BSA, HGS, MVO, HHO, and PSO.

Table 5 illustrates the outcomes of the comparison methods based on the fitness function’s mean and standard deviation. In comparison to other published methods, the proposed GTO-BSA got better results. On three datasets, it achieved the mean best results (NSL-KDD, CICID2017, and UNSW-NB15). The second significant indicator is that the HHO algorithm outperforms other algorithms in the BoT-IoT dataset of mean fitness function values. The proposed GTO-BSA algorithm is the second-best algorithm for this dataset. Another important aspect for evaluating algorithms’ performance is done by inspecting the STD value. Another important aspect of assessing algorithms’ performance is examining the STD value. A low STD value indicates that the algorithm achieved close values across multiple runs with a low distribution. GTO-BSA achieved the best STD value in three out of four datasets, indicating that GTO-BSA is a robust method for most datasets. 

In terms of accuracy, a comparison of accuracy results between GTO-BSA and other algorithms that are assessed in the same conditions are depicted in Table 6. The experimental results reveal that the GTO-BSA is superior in three out of four datasets, while HHO provided the best results on only the BoT-IoT dataset. It is important to highlight that GTO-BSA produced the second-best result on the BoT-IoT dataset. In comparison with the conventional GTO and BSA algorithms, the GTO-BSA also gave increased performance. The experimental results demonstrated that the proposed GTO-BSA method obtained the most informative features with higher accuracy values. The same conclusion was reached for sensitivity and specificity measures from Table 7 and Table 8, respectively.

Table 9 shows the numbers of selected features by the GTO-BSA and the competitor algorithms that treat the same datasets in terms of the average number of selected features. By examining the results of Table 9, we conclude that GTO-BSA achieved the best average number of selected features compared to other optimizers in two datasets out of four datasets used in this study. The HHO method obtained the best results in two datasets (UNSW-NB15 and BoT-IoT). By inspecting standard deviation, GTO-BSA is a robust method for most of the datasets relative to other methods.

The computational time of the comparing methods is shown in Table 10. The HGS method has the lowest computational time and is the fastest compared to the other methods. Because of its combined structure, the proposed GTO-BSA takes some time to discover the best solution; however, this type of problem does not require real-time execution.

**Figure 2:**
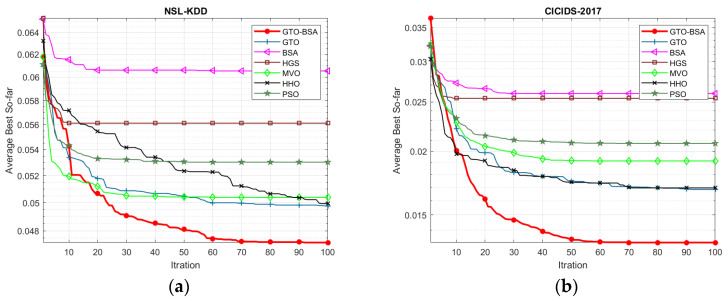

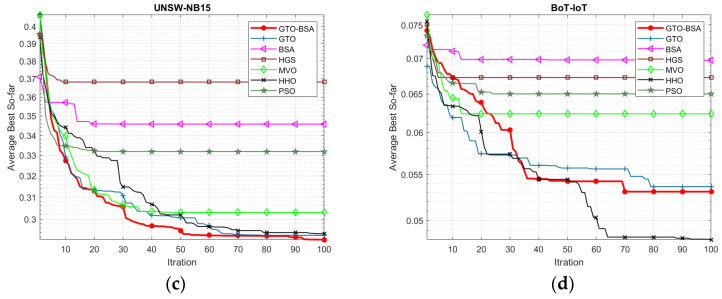
The convergence curves for the proposed algorithm and the other methods. (**a**) NSL-KDD; (**b**) CICID2017; (**c**) UNSW-NB15; (**d**) BoT-IoT.

Figure 2 depicts the convergence curve of the proposed GTO-BSA and its competitor algorithms using the four IoT-IDS datasets. The average value of objective functions acquired across 30 different trials of algorithms is used to plot these convergence curves. The x-axis shows the increase in iterations, while the y-axis displays the mean values of the goal function. It can be observed that the developed GTO-BSA method, which integrates the GTO and BSA, increases the rate of convergence towards optimal solutions.

**Figure 3:**
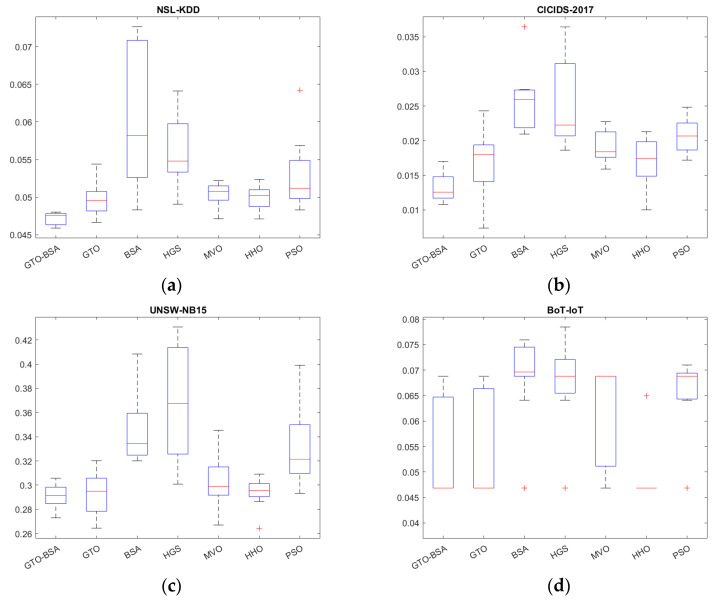
The boxplot for the proposed algorithm and the other methods. (**a**) NSL-KDD; (**b**) CICIDS-2017; (**c**) UNSE-NB15; (**d**) BoT-IoT.

The boxplot analysis can show the data distribution properties. Boxplots are graphical representations of data distributions in three quartiles: upper, lower, and middle. The algorithm’s minimum and maximum data points, which constitute the whisker’s edges, are the lowest and highest data points reached by the method. The ends of the rectangles define the lower and upper quartiles. There is a strong correlation between the data points if the boxplot is narrow. The boxplot of the competitive algorithms over four datasets is shown in Figure 3. In most of the datasets, the proposed GTO-BSA algorithm’s boxplots are highly narrow. They have the lowest values compared to the other techniques’ distributions.

Overall, it is clear from the results that the proposed GTO-BSA method performs much better than the original GTO algorithm in most of the used datasets. Through experiments, the GTO-BSA method proved its efficiency in solving feature selection problems.

## 6. Conclusions and Future Work

The specific nature of the Internet of Things (IoT) applications, which consist of millions of sensors, leads to generating a massive amount of data. Moreover, from these applications a critical issue arises in regard to guaranteeing the security and privacy of these data. In recent years, a number of security solutions based on machine learning (ML) have been presented, including intrusion detection systems (IDS). The presence of redundant or irrelevant data affects the performance of ML algorithms. This research aimed to present a novel feature selection (FS) approach by improving the performance of Gorilla Troops Optimizer (GTO) using the algorithm for bird swarms (BSA), which is named the GTO-BSA method. The performance of GTO was enhanced by adopting BSA, which has a strong ability to find the feasible regions that provide the best solution. The proposed GTO-BSA method’s performance was examined using four IoT-IDS datasets: NSL-KDD, CICIDS-2017, UNSW-NB15, and BoT-IoT, and compared with other competitive algorithms. Results from the experiments showed that the proposed GTO-BSA method produced superior outcomes against numerous current metaheuristic algorithms, including the original GTO and BSA, HGS, MVO, HHO, and PSO. It has achieved an accuracy of 95.5%, 98.7%, 81.5%, and 81.5% in the NSL-KDD, CICID2017, UNSW-NB, and BoT-IoT datasets, respectively. In future work, the efficiency of the proposed GTO-BSA method can be evaluated in different problems such as solving different multi-objective problems, ML hyperparameters optimization, and multilevel threshold segmentation.

## Figures and Tables

**Table 1:** Parameter settings.

Algorithms	Parameter	Values
PSO	Cognitive component $(c_{1}$)	2
	Social component $(c_{2}$)	2
	Inertia weight	0.2–0.9
BSA	Cognitive coefficient (*C*)	1.5
	Social accelerated coefficient (*S*)	1.5
	Positive constants *a*1 and *a*2	1
	Constant value (*P*)	[0.8, 1]
	Flowing factor (*FL*)	[0.5, 0.9]
	Flight behaviors (*FQ*)	3
MVO	Wormhole existence probability (WEP)	[0.2, 1]
	Traveling distance rate (TDR)	[0.6, 1]
HHO	Beta (*β*)	1.5
GTO	*β*	3
	*W*	0.8
	*p*	0.03
HGS	--	--
Common settings	Population size (N)	30
	Maximum number of iterations	100
	Number of independent runs	25
	Problem dimensions	Number of features

**Table 2:** NSL-KDD dataset attack types.

Attack Type	Train	Test
Normal	67,343	9710
DOS	45,927	7458
PRP	11,656	2422
R2L	995	2887
U2R	52	67
Total	125,973	22,544

**Table 3:** CIC-IDS2017 dataset attack types.

Attack Type	Train	Test
Benign	727,397	163,572
DDOS	112,901	25,388
FTP-Patator	6997	1574
PortScan	140,043	31,492
SSH-Patator	5201	1169
Web Attack Brute Force	1329	299
Web Attack XSS	575	129
Web Attack Sql Injection	19	4
Total	904,056	223,627

**Table 4:** BoT-IoT dataset attack types.

Attack Type	Train	Test
DDOS	112,901	25,388
DOS	1,320,148	330,112
Reconnaissance	72,919	18,163
Normal	370	107
Theft	65	14
Total	2,934,817	733,705

**Table 5:** The performance of the GTO-BSA against other competitors in terms of **fitness** in intrusion detection datasets.

	Measures	Algorithms						
		GTO-BSA	GTO	BSA	HGS	MVO	HHO	PSO
NSL-KDD	Mean	**0.047193**	0.049763	0.06053	0.056116	0.050386	0.049931	0.053031
	STD	**0.000862**	0.002386	0.009632	0.005113	0.001606	0.001654	0.005204
CICIDS-2017	Mean	**0.013246**	0.016835	0.025962	0.025408	0.019148	0.016953	0.020728
	STD	**0.002095**	0.005059	0.005019	0.006644	0.002374	0.003776	0.002628
UNSW-NB15	Mean	**0.290922**	0.292833	0.34572	0.368174	0.302974	0.293527	0.331793
	STD	**0.010444**	0.018691	0.031901	0.050562	0.023094	0.013572	0.034335
BoT-IoT	Mean	0.053071	0.053622	0.069689	0.067243	0.062369	**0.048044**	0.065001
	STD	0.009179	0.009984	0.007162	0.009116	0.009805	**0.004672**	0.007722

**Table 6:** The performance of the GTO-BSA against other competitors in terms of **accuracy** in intrusion detection datasets.

	Measures	Algorithms						
		GTO-BSA	GTO	BSA	HGS	MVO	HHO	PSO
NSL-KDD	Mean	**0.955964**	0.954293	0.944063	0.947906	0.95317	0.954399	0.950098
	STD	**0.000777**	0.00233	0.00952	0.005163	0.001516	0.001287	0.005462
CICIDS-2017	Mean	**0.987915**	0.985261	0.976738	0.978577	0.983993	0.985158	0.982494
	STD	**0.001997**	0.004112	0.004844	0.005967	0.002079	0.002972	0.002462
UNSW-NB15	Mean	**0.710138**	0.707246	0.654365	0.632616	0.697934	0.706394	0.669214
	STD	**0.010759**	0.018133	0.032073	0.050747	0.023381	0.012971	0.034122
BoT-IoT	Mean	0.948525	0.947912	0.932469	0.935108	0.939358	**0.953266**	0.937036
	STD	0.008635	0.009517	0.00701	0.00871	0.009511	**0.004285**	0.007442

**Table 7:** The performance of the GTO-BSA against other competitors in terms of **sensitivity** in intrusion detection datasets.

	Measures	Algorithms						
		GTO-BSA	GTO	BSA	HGS	MVO	HHO	PSO
NSL-KDD	Mean	**0.914219**	0.903207	0.900309	0.894513	0.898184	0.905912	0.88949
	STD	**0.006015**	0.015608	0.011713	0.021994	0.017995	0.012041	0.017191
CICIDS-2017	Mean	**0.972644**	0.961626	0.943389	0.955547	0.967705	0.967705	0.965805
	STD	**0.004299**	0.00974	0.014617	0.020866	0.005613	0.005613	0.009009
UNSW-NB15	Mean	**0.815385**	0.778846	0.751923	0.682692	0.780769	0.786538	0.773077
	STD	0.052656	0.041881	0.079914	0.08583	0.089989	**0.020865**	0.090738
BoT-IoT	Mean	0.992832	0.98853	0.951254	0.962007	0.967025	**0.999283**	0.964875
	STD	0.015024	0.019264	0.019005	0.02434	0.026485	**0.002776**	0.023511

**Table 8:** The performance of the GTO-BSA against other competitors in terms of **specificity** in intrusion detection datasets.

	Measures	Algorithms						
		GTO-BSA	GTO	BSA	HGS	MVO	HHO	PSO
NSL-KDD	Mean	0.97365	0.973865	0.970727	0.974295	0.974897	0.973306	**0.975585**
	STD	**0.001684**	0.003236	0.004379	0.004325	0.00297	0.001927	0.002112
CICIDS-2017	Mean	**0.996798**	0.965948	0.994022	0.994236	0.996798	0.996798	0.99605
	STD	0.001977	0.056202	0.003565	0.001977	0.001095	0.00135	**0.001014**
UNSW-NB15	Mean	0.877049	0.802766	0.867572	0.840164	**0.87833**	0.822234	0.866291
	STD	0.019192	**0.118543**	0.024195	0.050314	0.018843	0.102639	0.018255
BoT-IoT	Mean	**0.962278**	0.650047	0.65042	0.927731	0.85845	0.511111	0.928478
	STD	**0.134499**	0.2545	0.255047	0.183337	0.238603	0.135247	0.183633

**Table 9:** The performance of the GTO-BSA against other competitors in terms of the number of **selected features** in intrusion detection datasets.

	Measures	Algorithms						
		GTO-BSA	GTO	BSA	HGS	MVO	HHO	PSO
NSL-KDD	Mean	**14.75**	18.5	21.125	18.625	16.5	19.625	14.875
	STD	**1.752549**	3.162278	4.290771	2.263846	2.725541	3.583195	2.799872
CICIDS-2017	Mean	**10**	17.5	22.875	32.75	25.75	17.625	26.5
	STD	**2.390457**	8.124038	7.337526	6.670832	4.832923	7.386039	3.422614
UNSW-NB15	Mean	16.625	12.625	14.875	18.75	16.5	**12**	18.125
	STD	2.445842	4.274091	4.48609	3.654743	**2.203893**	5.606119	4.015595
BoT-IoT	Mean	2.533333	2.466667	3.4	3.6	2.8	**2.133333**	3.2
	STD	0.833809	0.743223	1.055597	1.121224	0.560612	**0.516398**	0.676123

**Table 10:** The performance of the GTO-BSA against other competitors in terms of **computational time** in intrusion detection datasets.

	Measures	Algorithms						
		GTO-BSA	GTO	BSA	HGS	MVO	HHO	PSO
NSL-KDD	Mean	10,205.83	9719.664	6515.84	**661.7222**	5441.159	12,476.16	4604.313
	STD	1531.406	2136.192	1670.182	**325.9167**	1879.629	2498.787	1889.04
CICIDS-2017	Mean	2270.918	6988.469	5067.099	**545.4723**	6531.616	8062.423	6678.273
	STD	221.0268	3199.149	1998.733	**132.8222**	1380.878	3804.885	1025.475
UNSW-NB15	Mean	161.2396	113.3642	74.8803	**12.57722**	77.87134	146.2428	80.89471
	STD	4.890585	9.515462	8.096915	10.47935	**3.576578**	18.83938	4.238727
BoT-IoT	Mean	145.7462	108.6355	68.978	**10.74187**	70.17324	144.7192	71.09266
	STD	4.535463	5.233262	6.166675	8.576165	**2.955785**	5.982527	3.687295

## Data Availability

The data are available online.

## References

[1] El-Hasnony I.M., Mostafa R.R., Elhoseny M., Barakat S.I. (2021). Leveraging Mist and Fog for Big Data Analytics in IoT Environment. Trans. Emerg. Telecommun. Technol..

[2] Lee I. (2020). Internet of Things (IoT) Cybersecurity: Literature Review and Iot Cyber Risk Management. Future Internet.

[3] Kushwah G.S., Ranga V. (2020). Voting Extreme Learning Machine Based Distributed Denial of Service Attack Detection in Cloud Computing. J. Inf. Secur. Appl..

[4] Louvieris P., Clewley N., Liu X. (2013). Effects-Based Feature Identification for Network Intrusion Detection. Neurocomputing.

[5] Al-Jarrah O.Y., Alhussein O., Yoo P.D., Muhaidat S., Taha K., Kim K. (2016). Data Randomization and Cluster-Based Partitioning for Botnet Intrusion Detection. IEEE Trans. Cybern..

[6] Ashraf J., Keshk M., Moustafa N., Abdel-Basset M., Khurshid H., Bakhshi A.D., Mostafa R.R. (2021). IoTBoT-IDS: A Novel Statistical Learning-Enabled Botnet Detection Framework for Protecting Networks of Smart Cities. Sustain. Cities Soc..

[7] Zhou Y., Cheng G., Jiang S., Dai M. (2020). Building an Efficient Intrusion Detection System Based on Feature Selection and Ensemble Classifier. Comput. Netw..

[8] Wang K., Du M., Maharjan S., Sun Y. (2017). Strategic Honeypot Game Model for Distributed Denial of Service Attacks in the Smart Grid. IEEE Trans. Smart Grid.

[9] Wang K., Du M., Sun Y., Vinel A., Zhang Y. (2016). Attack Detection and Distributed Forensics in Machine-to-Machine Networks. IEEE Netw..

[10] Wang K., Du M., Yang D., Zhu C., Shen J., Zhang Y. (2016). Game-Theory-Based Active Defense for Intrusion Detection in Cyber-Physical Embedded Systems. ACM Trans. Embed. Comput. Syst..

[11] De la Hoz E., de la Hoz E., Ortiz A., Ortega J., Prieto B. (2015). PCA Filtering and Probabilistic SOM for Network Intrusion Detection. Neurocomputing.

[12] Du M., Wang K., Chen Y., Wang X., Sun Y. (2018). Big Data Privacy Preserving in Multi-Access Edge Computing for Heterogeneous Internet of Things. IEEE Commun. Mag..

[13] Du M., Wang K., Xia Z., Zhang Y. (2018). Differential Privacy Preserving of Training Model in Wireless Big Data with Edge Computing. IEEE Trans. Big Data.

[14] Mishra P., Varadharajan V., Tupakula U., Pilli E.S. (2019). A Detailed Investigation and Analysis of Using Machine Learning Techniques for Intrusion Detection. IEEE Commun. Surv. Tutor..

[15] Aljawarneh S., Aldwairi M., Yassein M.B. (2018). Anomaly-Based Intrusion Detection System through Feature Selection Analysis and Building Hybrid Efficient Model. J. Comput. Sci..

[16] Ambusaidi M.A., He X., Nanda P., Tan Z. (2016). Building an Intrusion Detection System Using a Filter-Based Feature Selection Algorithm. IEEE Trans. Comput..

[17] Guyon I., Elisseeff A. (2003). An introduction to variable and feature selection. J. Mach. Learn. Res..

[18] Xue B., Zhang M., Browne W.N., Yao X. (2016). A Survey on Evolutionary Computation Approaches to Feature Selection. IEEE Trans. Evol. Comput..

[19] El-Hasnony I.M., Barakat S.I., Elhoseny M., Mostafa R.R. (2020). Improved Feature Selection Model for Big Data Analytics. IEEE Access.

[20] Nguyen M.T., Kim K. (2020). Genetic Convolutional Neural Network for Intrusion Detection Systems. Future Gener. Comput. Syst..

[21] Gauthama Raman M.R., Somu N., Kirthivasan K., Liscano R., Shankar Sriram V.S. (2017). An Efficient Intrusion Detection System Based on Hypergraph—Genetic Algorithm for Parameter Optimization and Feature Selection in Support Vector Machine. Knowl. Based Syst..

[22] Malhotra S., Bali V., Paliwal K.K. (2017). Genetic programming and K-Nearest neighbour classifier based intrusion detection model. Proceedings of the 2017 7th International Conference on Cloud Computing, Data Science & Engineering-Confluence.

[23] Ghosh P., Karmakar A., Sharma J., Phadikar S. (2019). CS-PSO based intrusion detection system in cloud environment. Emerging Technologies in Data Mining and Information Security.

[24] Seth J.K., Chandra S. (2018). MIDS: Metaheuristic based intrusion detection system for cloud using k-NN and MGWO. Proceedings of the International Conference on Advances in Computing and Data Sciences.

[25] RM S.P., Maddikunta P.K.R., Parimala M., Koppu S., Gadekallu T.R., Chowdhary C.L., Alazab M. (2020). An Effective Feature Engineering for DNN Using Hybrid PCA-GWO for Intrusion Detection in IoMT Architecture. Comput. Commun..

[26] Mayuranathan M., Murugan M., Dhanakoti V. (2021). Best Features Based Intrusion Detection System by RBM Model for Detecting DDoS in Cloud Environment. J. Ambient Intell. Humaniz. Comput..

[27] Ewees A.A., Mostafa R.R., Ghoniem R.M., Gaheen M.A. (2022). Improved seagull optimization algorithm using Lévy flight and mutation operator for feature selection. Neural Comput. Appl..

[28] Del Ser J., Osaba E., Molina D., Yang X.S., Salcedo-Sanz S., Camacho D., Das S., Suganthan P.N., Coello Coello C.A., Herrera F. (2019). Bio-Inspired Computation: Where We Stand and What’s Next. Swarm Evol. Comput..

[29] Wolpert D.H., Macready W.G. (1997). No Free Lunch Theorems for Optimization. IEEE Trans. Evol. Comput..

[30] Abdollahzadeh B., Soleimanian Gharehchopogh F., Mirjalili S. (2021). Artificial Gorilla Troops Optimizer: A New Nature-Inspired Metaheuristic Algorithm for Global Optimization Problems. Int. J. Intell. Syst..

[31] Meng X.B., Gao X.Z., Lu L., Liu Y., Zhang H. (2016). A New Bio-Inspired Optimisation Algorithm: Bird Swarm Algorithm. J. Exp. Theor. Artif. Intell..

[32] Sayed G.I., Hassanien A.E. (2021). A novel chaotic artificial Gorilla Troops Optimizer and its application for fundus images segmentation. Proceedings of the International Conference on Advanced Intelligent Systems and Informatics.

[33] Cinar C. (2022). A Hybrid artificial differential evolution Gorilla Troops Optimizer for high-dimensional optimization problems. Differential Evolution: From Theory to Practice.

[34] Xiang L., Deng Z., Hu A. (2019). Forecasting Short-Term Wind Speed Based on IEWT-LSSVM Model Optimized by Bird Swarm Algorithm. IEEE Access.

[35] Aljarah I., Faris H., Mirjalili S., Al-Madi N., Sheta A., Mafarja M. (2019). Evolving Neural Networks Using Bird Swarm Algorithm for Data Classification and Regression Applications. Clust. Comput..

[36] Miramontes I., Guzman J.C., Melin P., Prado-Arechiga G. (2018). Optimal Design of Interval Type-2 Fuzzy Heart Rate Level Classification Systems Using the Bird Swarm Algorithm. Algorithms.

[37] Wang S., Liu S., Che X., Wang Z., Zhang J., Kong D. (2020). Recognition of Polycyclic Aromatic Hydrocarbons Using Fluorescence Spectrometry Combined with Bird Swarm Algorithm Optimization Support Vector Machine. Spectrochim. Acta Part A: Mol. Biomol. Spectrosc..

[38] Parashar M., Rajput S., Dubey H.M., Pandit M. (2017). Optimization of benchmark functions using a nature inspired Bird Swarm Algorithm. Proceedings of the 2017 3rd International Conference on Computational Intelligence & Communication Technology (CICT).

[39] Ismail F.H., Houssein E.H., Hassanien A.E. (2018). Chaotic bird swarm optimization algorithm. Proceedings of the International Conference on Advanced Intelligent Systems and Informatics.

[40] Wu D., Gao H. (2018). Multi-objective Bird Swarm Algorithm. Proceedings of the International Symposium on Artificial Intelligence and Robotics.

[41] Houssein E.H., Ahmed M.M., Abd Elaziz M., Ewees A.A., Ghoniem R.M. (2021). Solving Multi-Objective Problems Using Bird Swarm Algorithm. IEEE Access.

[42] Pruthi J., Arora S., Khanna K. (2018). Modified Bird Swarm Algorithm for Edge Detection in Noisy Images Using Fuzzy Reasoning. Comput. Methods Biomech. Biomed. Eng. Imaging Vis..

[43] Bhardwaj J., Nayak A. (2022). Medical image fusion using lifting wavelet and fractional bird swarm optimization. Proceedings of the International e-Conference on Intelligent Systems and Signal Processing.

[44] Pruthi J., Arora S., Khanna K. (2020). Segmentation of blood vessels from retinal fundus images using Bird Swarm Algorithm and river formation dynamics algorithm. Proceedings of the International Conference on Intelligent Computing and Smart Communication 2019.

[45] Mishra K., Majhi S.K. (2021). A Binary Bird Swarm Optimization Based Load Balancing Algorithm for Cloud Computing Environment. Open Comput. Sci..

[46] Kennedy J., Eberhart R. Particle swarm optimization. Proceedings of the ICNN’95-International Conference on Neural Networks.

[47] Moradi P., Gholampour M. (2016). A Hybrid Particle Swarm Optimization for Feature Subset Selection by Integrating a Novel Local Search Strategy. Appl. Soft Comput. J..

[48] Mistry K., Zhang L., Neoh S.C., Lim C.P., Fielding B. (2017). A Micro-GA Embedded PSO Feature Selection Approach to Intelligent Facial Emotion Recognition. IEEE Trans. Cybern..

[49] Chen K., Zhou F.Y., Yuan X.F. (2019). Hybrid Particle Swarm Optimization with Spiral-Shaped Mechanism for Feature Selection. Expert Syst. Appl..

[50] Mafarja M., Aljarah I., Faris H., Hammouri A.I., Al-Zoubi A.M., Mirjalili S. (2019). Binary Grasshopper Optimisation Algorithm Approaches for Feature Selection Problems. Expert Syst. Appl..

[51] Mafarja M., Aljarah I., Heidari A.A., Hammouri A.I., Faris H., Al-Zoubi A.M., Mirjalili S. (2018). Evolutionary Population Dynamics and Grasshopper Optimization Approaches for Feature Selection Problems. Knowl.-Based Syst..

[52] Mirjalili S., Gandomi A.H., Mirjalili S.Z., Saremi S., Faris H., Mirjalili S.M. (2017). Salp Swarm Algorithm: A Bio-Inspired Optimizer for Engineering Design Problems. Adv. Eng. Softw..

[53] Faris H., Mafarja M.M., Heidari A.A., Aljarah I., Al-Zoubi A.M., Mirjalili S., Fujita H. (2018). An Efficient Binary Salp Swarm Algorithm with Crossover Scheme for Feature Selection Problems. Knowl.-Based Syst..

[54] Aljarah I., Habib M., Faris H., Al-Madi N., Heidari A.A., Mafarja M., Elaziz M.A., Mirjalili S. (2020). A Dynamic Locality Multi-Objective Salp Swarm Algorithm for Feature Selection. Comput. Ind. Eng..

[55] Tubishat M., Idris N., Shuib L., Abushariah M.A.M., Mirjalili S. (2020). Improved Salp Swarm Algorithm Based on Opposition Based Learning and Novel Local Search Algorithm for Feature Selection. Expert Syst. Appl..

[56] Mirjalili S. (2016). SCA: A Sine Cosine Algorithm for Solving Optimization Problems. Knowl.-Based Syst..

[57] Neggaz N., Ewees A.A., Elaziz M.A., Mafarja M. (2020). Boosting Salp Swarm Algorithm by Sine Cosine Algorithm and Disrupt Operator for Feature Selection. Expert Syst. Appl..

[58] Kumar L., Bharti K.K. (2021). A Novel Hybrid BPSO–SCA Approach for Feature Selection. Nat. Comput..

[59] Hans R., Kaur H. (2020). Hybrid Binary Sine Cosine Algorithm and Ant Lion Optimization (SCALO) Approaches for Feature Selection Problem. Int. J. Comput. Mater. Sci. Eng..

[60] Zervoudakis K., Tsafarakis S. (2020). A Mayfly Optimization Algorithm. Comput. Ind. Eng..

[61] Bhattacharyya T., Chatterjee B., Singh P.K., Yoon J.H., Geem Z.W., Sarkar R. (2020). Mayfly in Harmony: A New Hybrid Meta-Heuristic Feature Selection Algorithm. IEEE Access.

[62] Kannan A., Maguire G.Q., Sharma A., Schoo P. Genetic algorithm based feature selection algorithm for effective intrusion detection in cloud networks. Proceedings of the 12th IEEE International Conference on Data Mining Workshops (ICDMW 2012).

[63] Nazir A., Khan R.A. (2021). A Novel Combinatorial Optimization Based Feature Selection Method for Network Intrusion Detection. Comput. Secur..

[64] Yang Y., Chen H., Heidari A.A., Gandomi A.H. (2021). Hunger games search: Visions, conception, implementation, deep analysis, perspectives, and towards performance shifts. Expert Syst. Appl..

[65] Mirjalili S., Mirjalili S.M., Hatamlou A. (2016). Multi-verse optimizer: A nature-inspired algorithm for global optimization. Neural Comput..

[66] Heidari A.A., Mirjalili S., Faris H., Aljarah I., Mafarja M., Chen H. (2019). Harris hawks optimization: Algorithm and applications. Future Gener. Comput. Syst..

[67] Tavallaee M., Bagheri E., Lu W., Ghorbani A.A. (2009). A detailed analysis of the KDD CUP 99 data set. Proceedings of the 2009 IEEE Symposium on Computational Intelligence for Security and Defense Applications.

[68] Rosset S., Inger A. (2000). KDD-cup 99: Knowledge discovery in a charitable organization’s donor database. ACM SIGKDD Explor. Newsl..

[69] Sharafaldin I., Lashkari A.H., Ghorbani A.A. (2018). Toward generating a new intrusion detection dataset and intrusion traffic characterization. ICISSp.

[70] Moustafa N., Slay J. (2015). UNSW-NB15: A comprehensive data set for network intrusion detection systems (UNSW-NB15 network data set). Proceedings of the 2015 Military Communications and Information Systems Conference (MilCIS).

[71] Koroniotis N., Moustafa N., Sitnikova E., Turnbull B. (2019). Towards the development of realistic botnet dataset in the internet of things for network forensic analytics: Bot-IoT dataset. Future Gener. Comput. Syst..

